# Supplementary material for: Rapid geomagnetic changes inferred from Earth observations and numerical simulations
Source: Nat Commun. 2020 Jul 6;11:3371. doi: 10.1038/s41467-020-16888-0 (PMC7338531; doi:10.1038/s41467-020-16888-0)
Supplement: Supplementary file 1 — Supplementary Information [file 41467_2020_16888_MOESM1_ESM.pdf]

Supplementary Information for “Rapid geomagnetic changes inferred from Earth observations and numerical simulations”

Christopher J. Davies[1] & Catherine G. Constable[2]

[1] *School of Earth and Environment, University of Leeds, Leeds LS2 9JT, UK*

[2] *Institute of Geophysics and Planetary Physics, Scripps Institution of Oceanography, University of California at San Diego, La Jolla, CA, 92093-0225, USA*

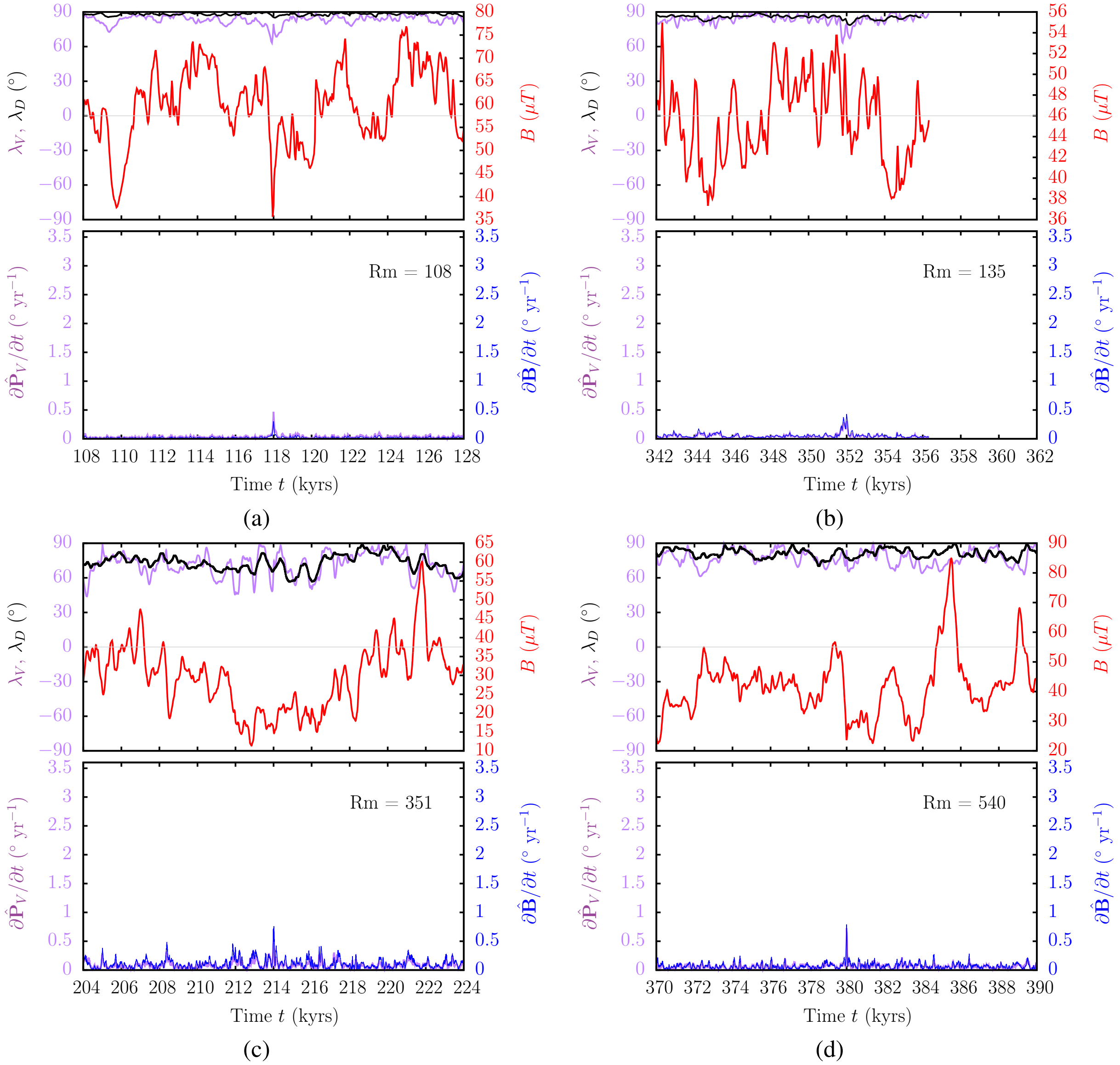

λ_V, λ_D (°)
B (μT)
$\partial \hat{\mathbf{P}}_V / \partial t$ (° yr^{-1})
$\partial \hat{\mathbf{B}} / \partial t$ (° yr^{-1})
Rm = 108
Time t (kyrs)
(a)
Rm = 135
Time t (kyrs)
(b)
Rm = 351
Time t (kyrs)
(c)
Rm = 540
Time t (kyrs)
(d)

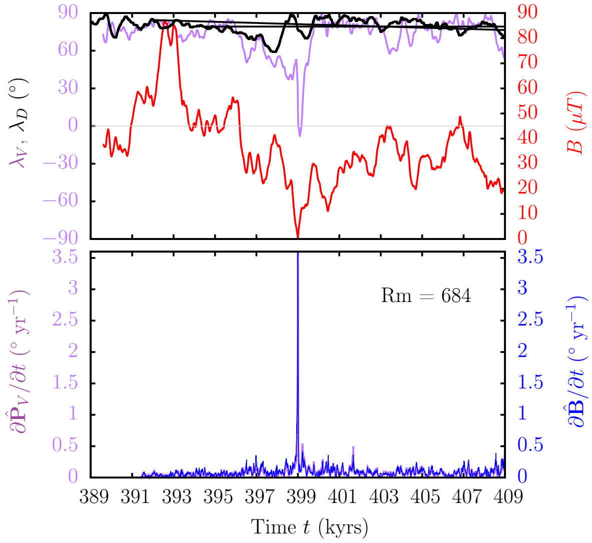

λ_V, λ_D (°)
B (μT)
$\partial\hat{\mathbf{P}}_V/\partial t$ (° yr^{-1})
$\partial\hat{\mathbf{B}}/\partial t$ (° yr^{-1})
Rm = 684
Time t (kyrs)

(e)

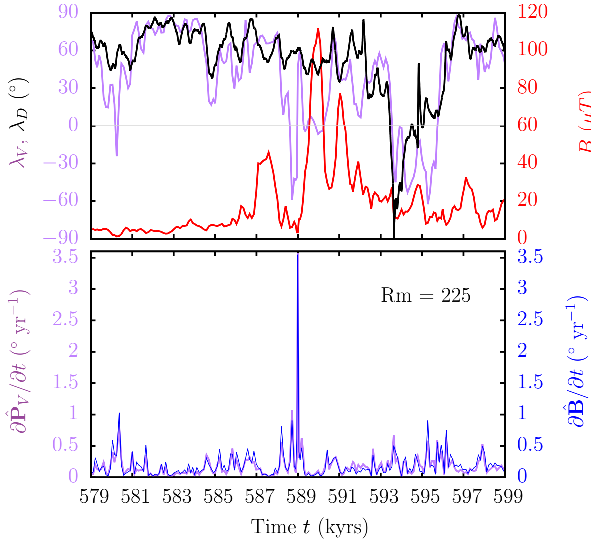

λ_V, λ_D (°)
B (μT)
$\partial\hat{\mathbf{P}}_V/\partial t$ (° yr^{-1})
$\partial\hat{\mathbf{B}}/\partial t$ (° yr^{-1})
Rm = 225
Time t (kyrs)

(f)

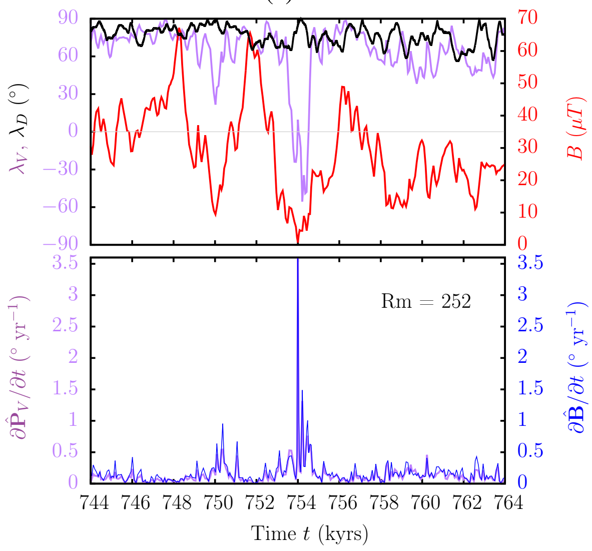

λ_V, λ_D (°)
B (μT)
$\partial\hat{\mathbf{P}}_V/\partial t$ (° yr^{-1})
$\partial\hat{\mathbf{B}}/\partial t$ (° yr^{-1})
Rm = 252
Time t (kyrs)

(g)

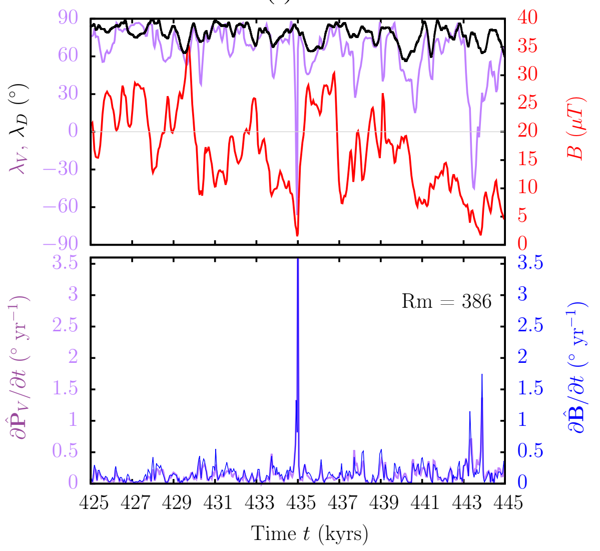

λ_V, λ_D (°)
B (μT)
$\partial\hat{\mathbf{P}}_V/\partial t$ (° yr^{-1})
$\partial\hat{\mathbf{B}}/\partial t$ (° yr^{-1})
Rm = 386
Time t (kyrs)

(h)

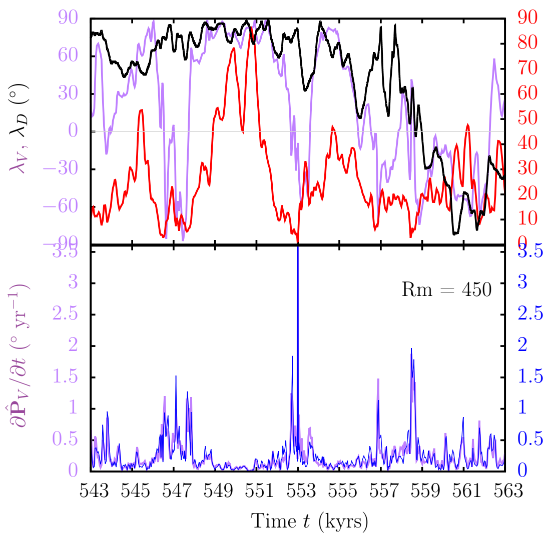

λ_V, λ_D (°)
B (μT)
$\partial\hat{\mathbf{P}}_V/\partial t$ (° yr^{-1})
$\partial\hat{\mathbf{B}}/\partial t$ (° yr^{-1})
Rm = 450
Time t (kyrs)

(i)

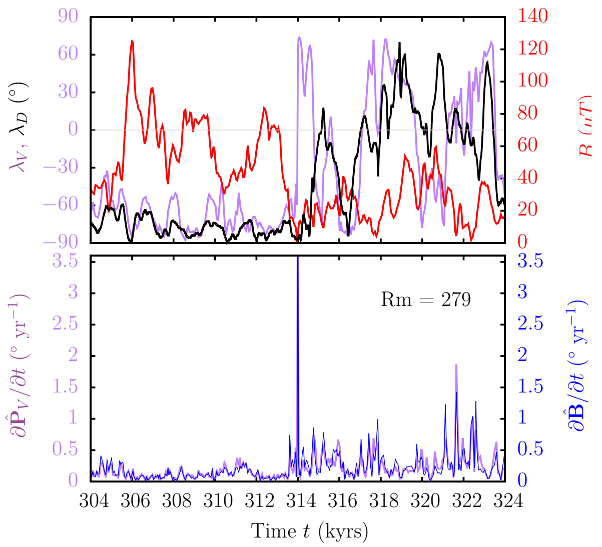

λ_V, λ_D (°)
B (μT)
$\partial\hat{\mathbf{P}}_V/\partial t$ (° yr^{-1})
$\partial\hat{\mathbf{B}}/\partial t$ (° yr^{-1})
Rm = 279
Time t (kyrs)

(j)

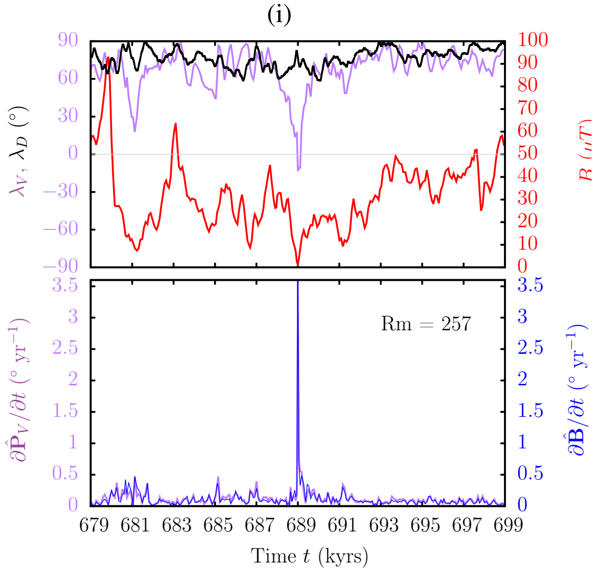

λ_V, λ_D (°)
B (μT)
$\partial\hat{\mathbf{P}}_V/\partial t$ (° yr^{-1})
$\partial\hat{\mathbf{B}}/\partial t$ (° yr^{-1})
Rm = 257
Time t (kyrs)

(k)

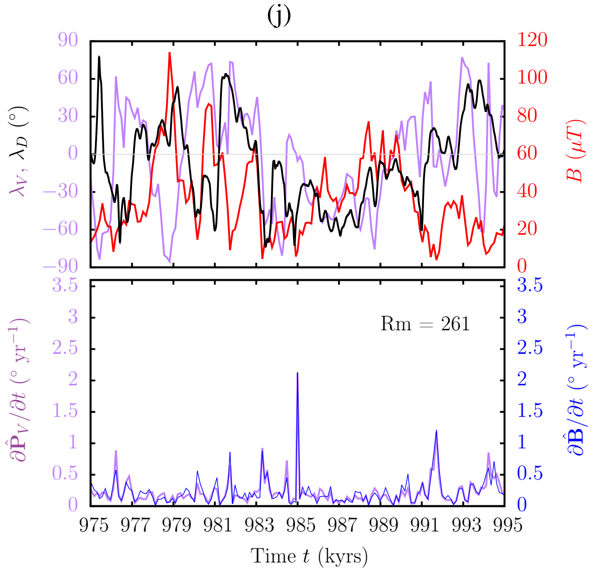

λ_V, λ_D (°)
B (μT)
$\partial\hat{\mathbf{P}}_V/\partial t$ (° yr^{-1})
$\partial\hat{\mathbf{B}}/\partial t$ (° yr^{-1})
Rm = 261
Time t (kyrs)

(l)

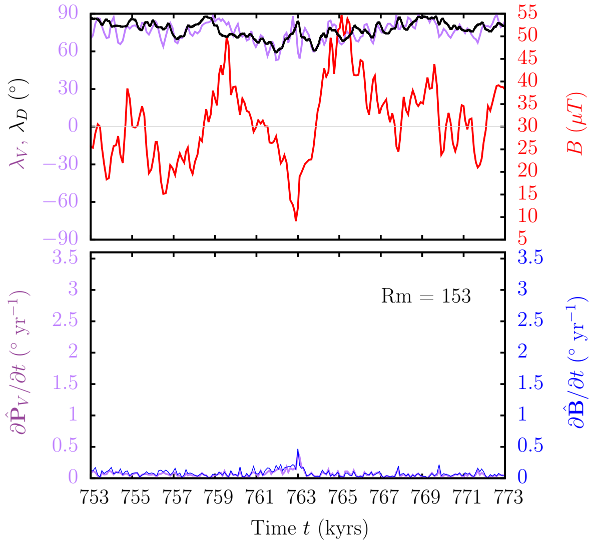

(m)

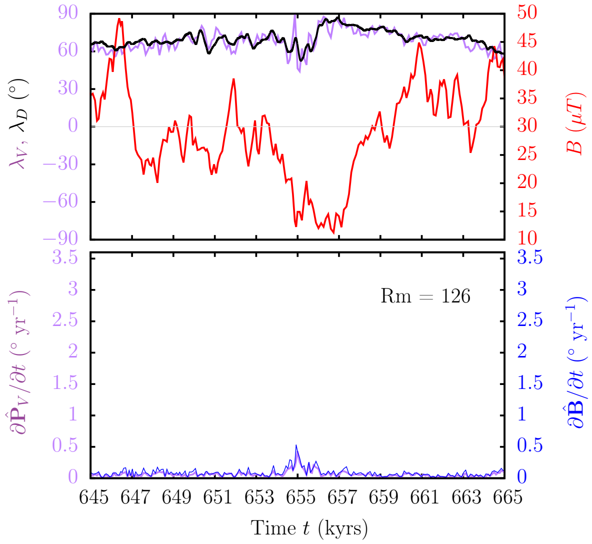

(n)

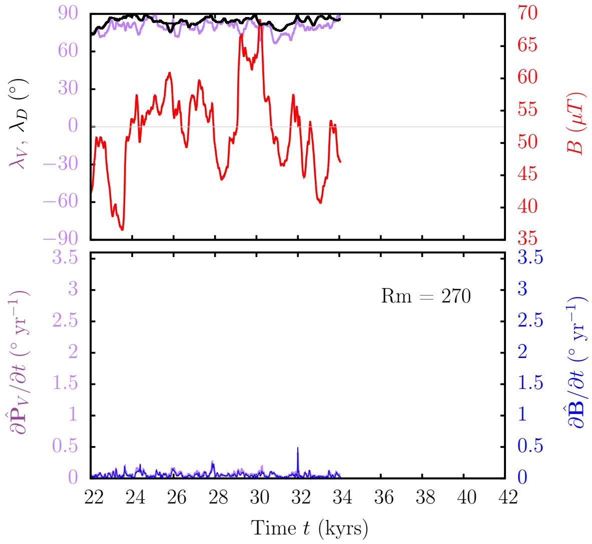

(o)

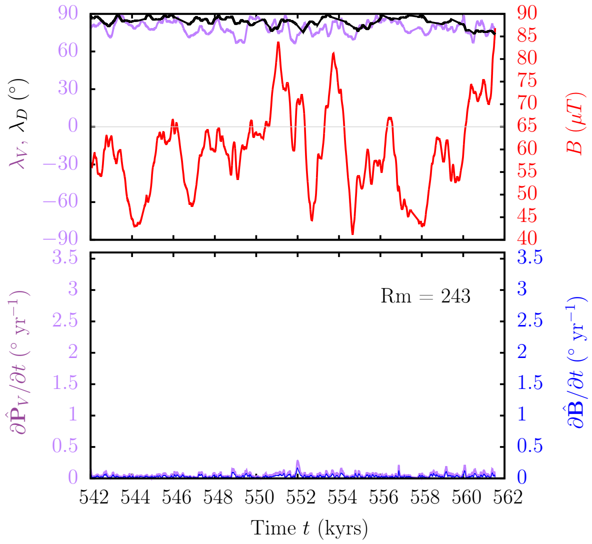

(p)

Supplementary Figure 1: Time-series of directional and intensity data at the locations of the maximum rates of VGP change, $(\partial\hat{\mathbf{P}}_V/\partial t)_{\rm ex}$. The top row of each panel shows the latitude λ_V of the VGP $\hat{\mathbf{P}}_V$ (purple), the dipole latitude λ_D (black), and the field strength B (red). The bottom row shows $\partial\hat{\mathbf{P}}_V/\partial t$ (purple) and the rate of change of the field vector $\hat{\mathbf{B}}$, $\partial\hat{\mathbf{B}}/\partial t$ (blue). The 20 thousand year focus for the figures is chosen with the most extreme rate of directional change as the mid-point. The magnetic Reynolds number is denoted Rm.

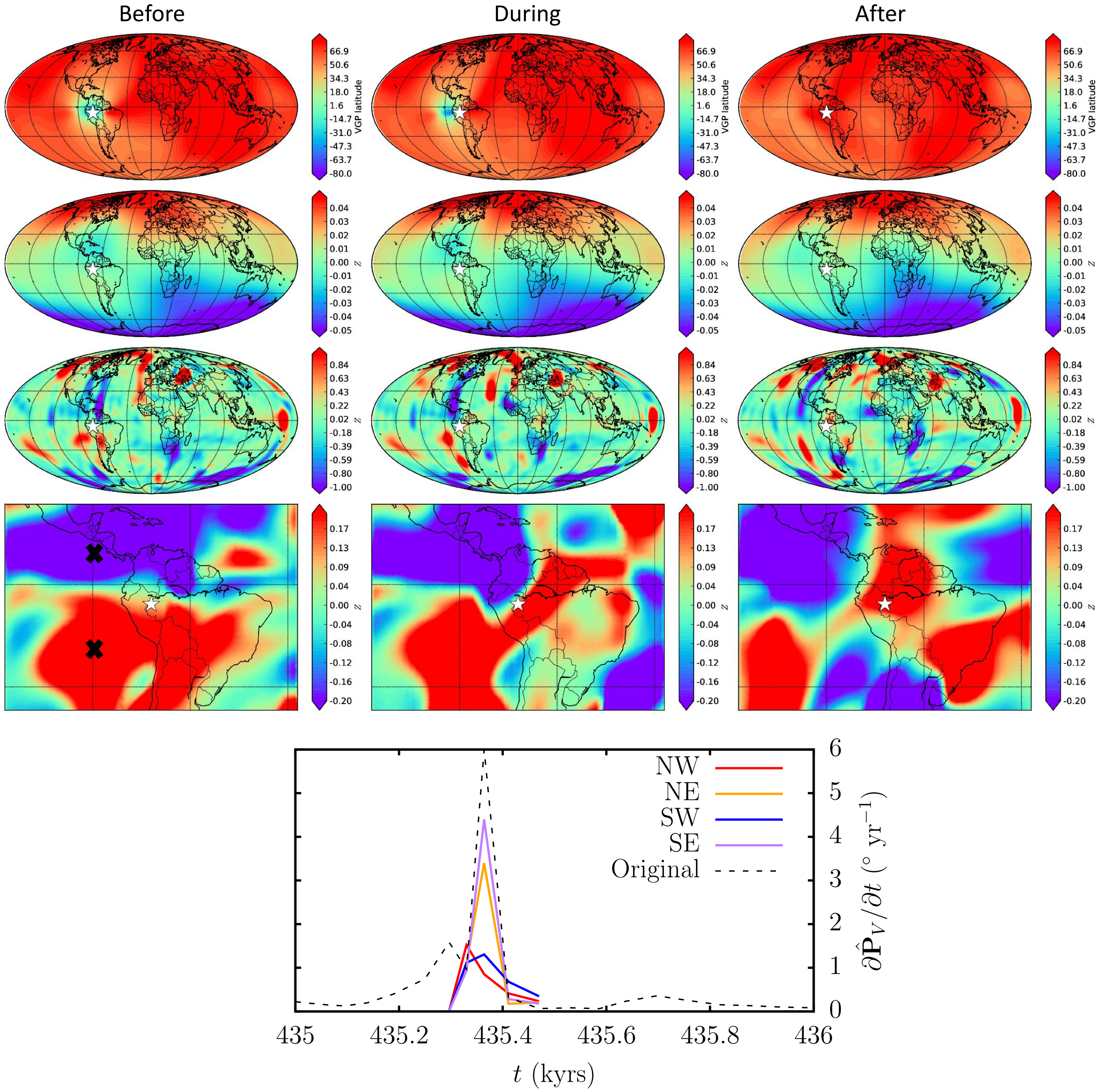

Supplementary Figure 2: Evolution of an extreme change in $\hat{\mathbf{P}}_V$ for the simulation with $E = 5 \times 10^{-4}$, $Pm = 10$, $Ra = 250$ and $Rm = 386$. From top to bottom the rows show the VGP latitude λ_V, Mollweide projections of the vertical component of the magnetic field Z at the surface and CMB and a local Mercator projection of the CMB field in the region of maximum directional change. Columns show times just before (left), during (center), and just after (right) the extreme change (white star). The largest effect on $\partial\hat{\mathbf{P}}_V/\partial t$ comes from the two reversed flux patches marked by crosses (bottom panel). The extreme event occurs when the null flux line separating the two reversed regions moves under the site (since the site is almost on the equator there is little influence of the dipole field).

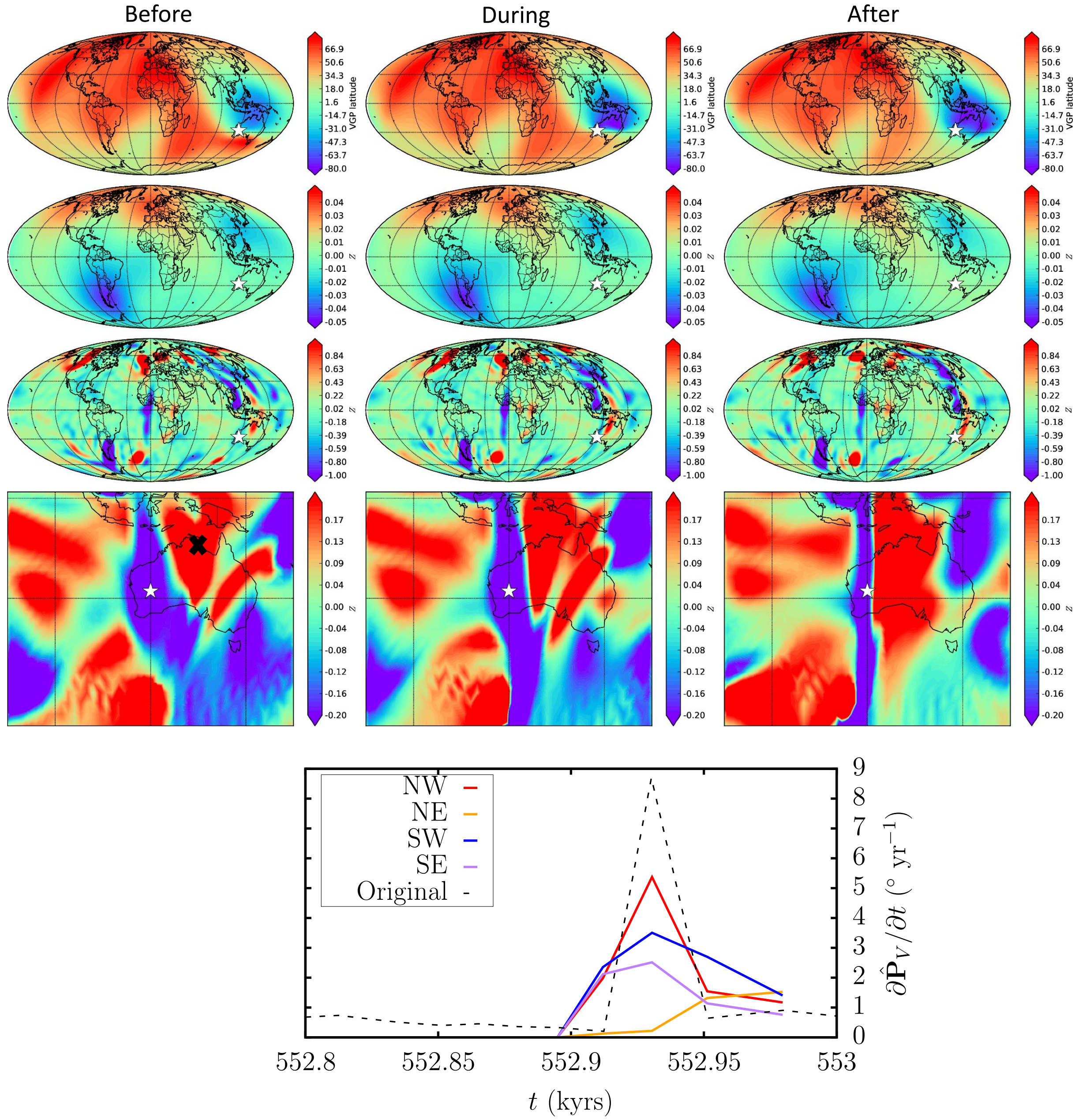

Supplementary Figure 3: Evolution of an extreme change in $\hat{\mathbf{P}}_V$ for the simulation with $E = 5 \times 10^{-4}$, $Pm = 10$, $Ra = 350$ and $Rm = 450$. From top to bottom the rows show the VGP latitude λ_V, Mollweide projections of the vertical component of the magnetic field Z at the surface and CMB and a local Mercator projection of the CMB field in the region of maximum directional change. Columns show times just before (left), during (center), and just after (right) the extreme change (white star). The largest contribution to $\partial\hat{\mathbf{P}}_V/\partial t$ comes from the reversed flux patch marked by a cross that occupies north-west (NW) and north-east (NE) quadrants (see bottom panel).

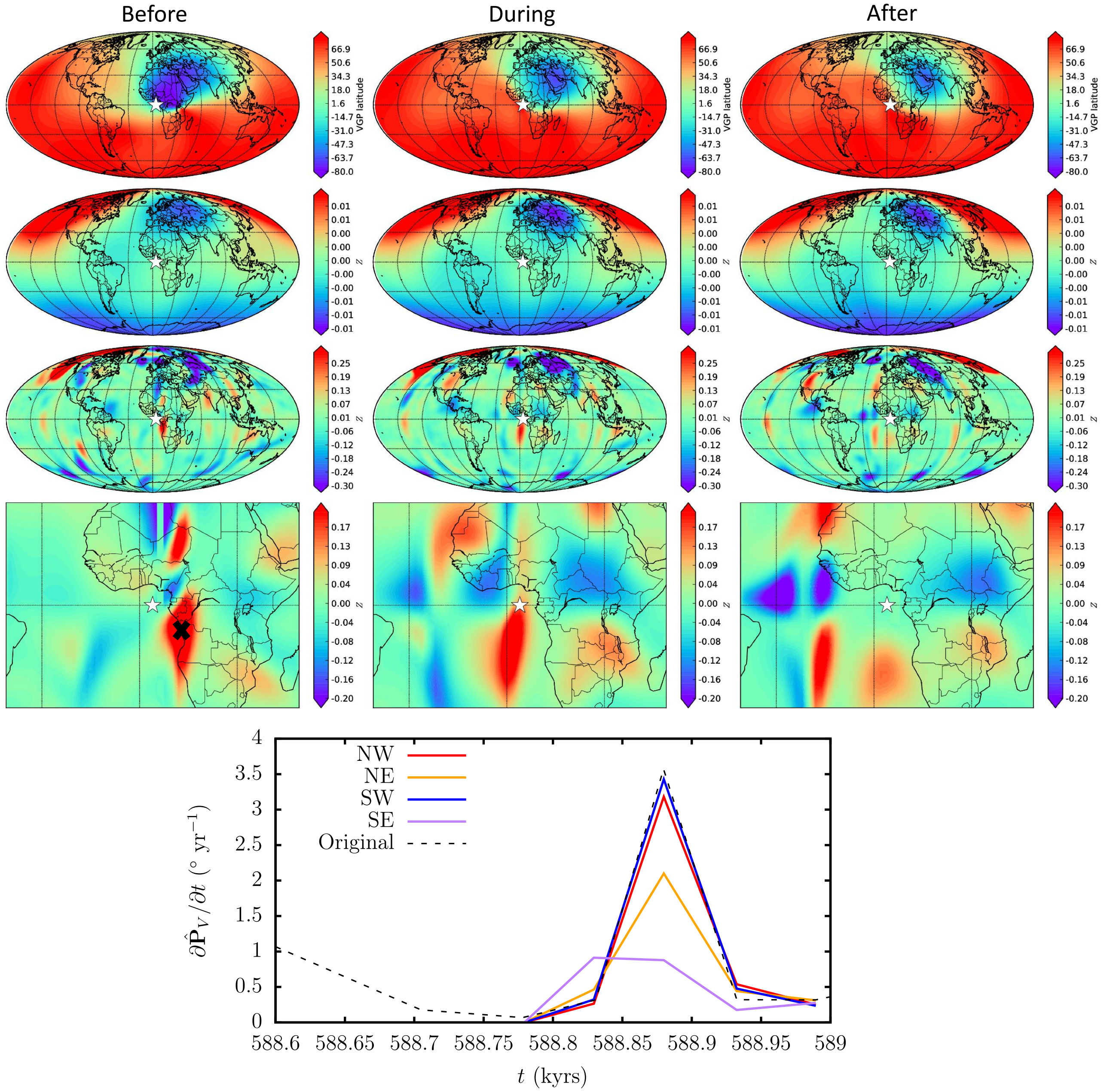

Supplementary Figure 4: Evolution of an extreme change in $\hat{\mathbf{P}}_V$ for the simulation with $E = 5 \times 10^{-4}$, $Pm = 5$, $Ra = 250$ and $Rm = 225$. From top to bottom the rows show the VGP latitude λ_V, Mollweide projections of the vertical component of the magnetic field Z at the surface and CMB and a local Mercator projection of the CMB field in the region of maximum directional change. Columns show times just before (left), during (center), and just after (right) the extreme change (white star). The largest contribution to $\partial\hat{\mathbf{P}}_V/\partial t$ comes from the patch of reversed flux marked by a cross in the south-east (SE) quadrant (see bottom panel). The extreme event occurs when the null flux line separating reversed flux patches on either side of the equator passes below the site (note there is little contribution from the axial dipole at this location).

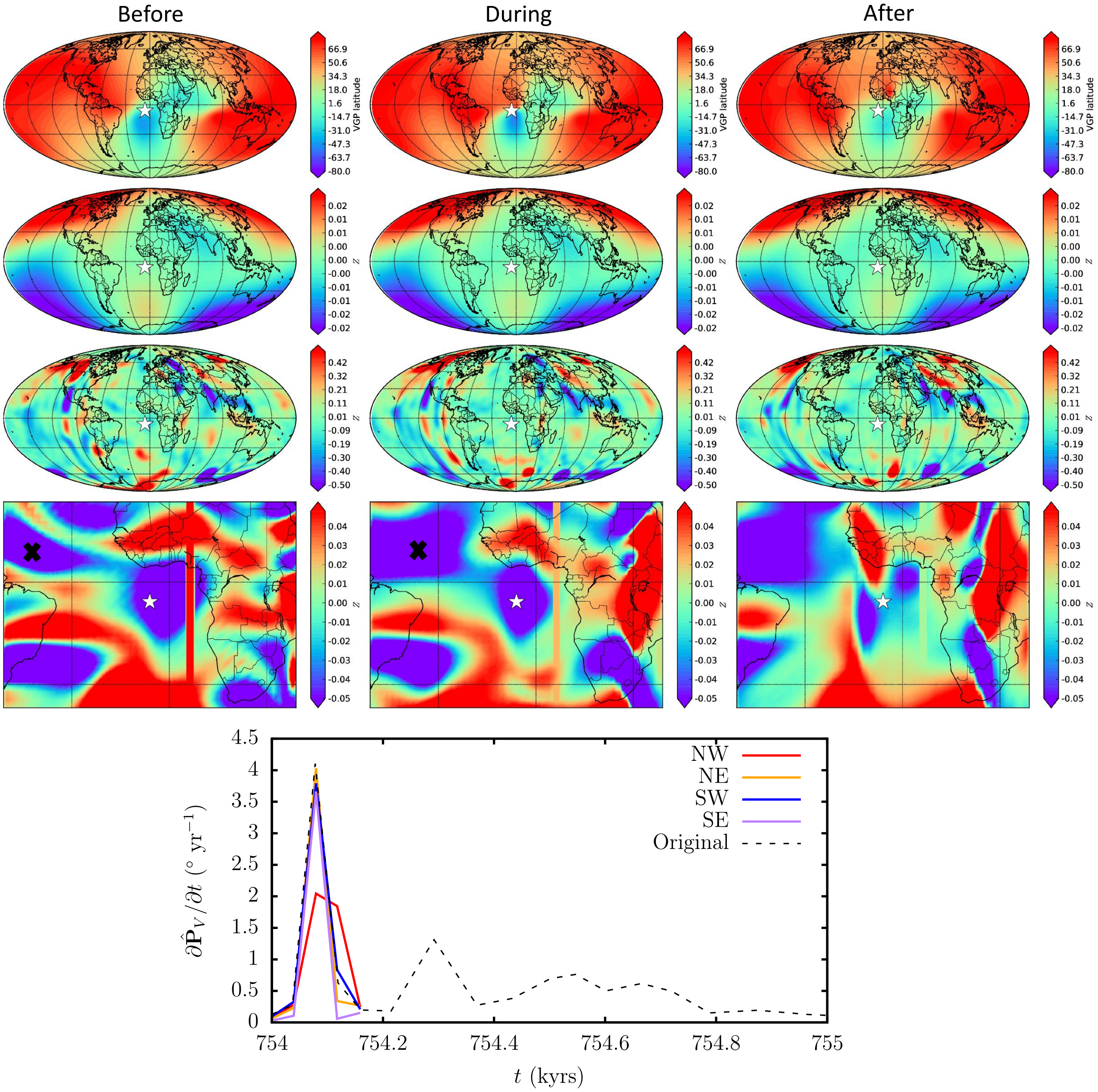

Supplementary Figure 5: Evolution of an extreme change in $\hat{\mathbf{P}}_V$ for the simulation with $E = 5 \times 10^{-4}$, $Pm = 5$, $Ra = 350$ and $Rm = 252$. From top to bottom the rows show the VGP latitude λ_V, Mollweide projections of the vertical component of the magnetic field Z at the surface and CMB and a local Mercator projection of the CMB field in the region of maximum directional change. Columns show times just before (left), during (center), and just after (right) the extreme change (white star). The largest contribution to $\partial\hat{\mathbf{P}}_V/\partial t$ comes from the reversed flux patch marked with a cross in the north-west (BW) quadrant (see bottom panel), which moves towards the location of maximum change.

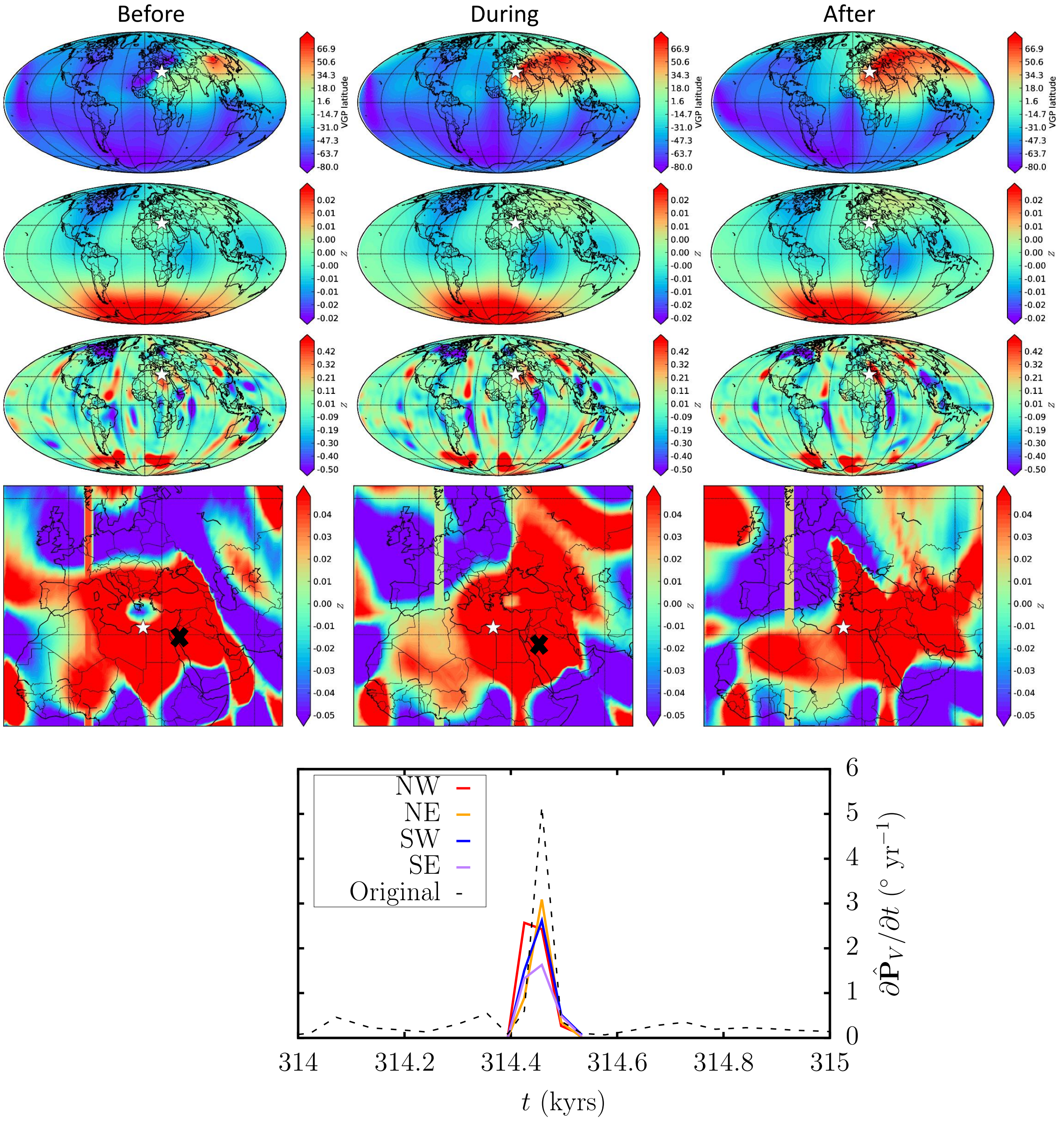

Supplementary Figure 6: Evolution of an extreme change in $\hat{\mathbf{P}}_V$ for the simulation with $E = 5 \times 10^{-4}$, $Pm = 5$, $Ra = 450$ and $Rm = 279$. From top to bottom the rows show the VGP latitude λ_V, Mollweide projections of the vertical component of the magnetic field Z at the surface and CMB and a local Mercator projection of the CMB field in the region of maximum directional change. Columns show times just before (left), during (center), and just after (right) the extreme change (white star). A large reversed patch (cross) lies directly beneath the star and all quadrants contribute to the rapid change; the largest contribution is from the southeastern quadrant that contains the strongest reversed flux. Note also that the weakest contribution comes from the quadrant with the least reversed flux (NE).

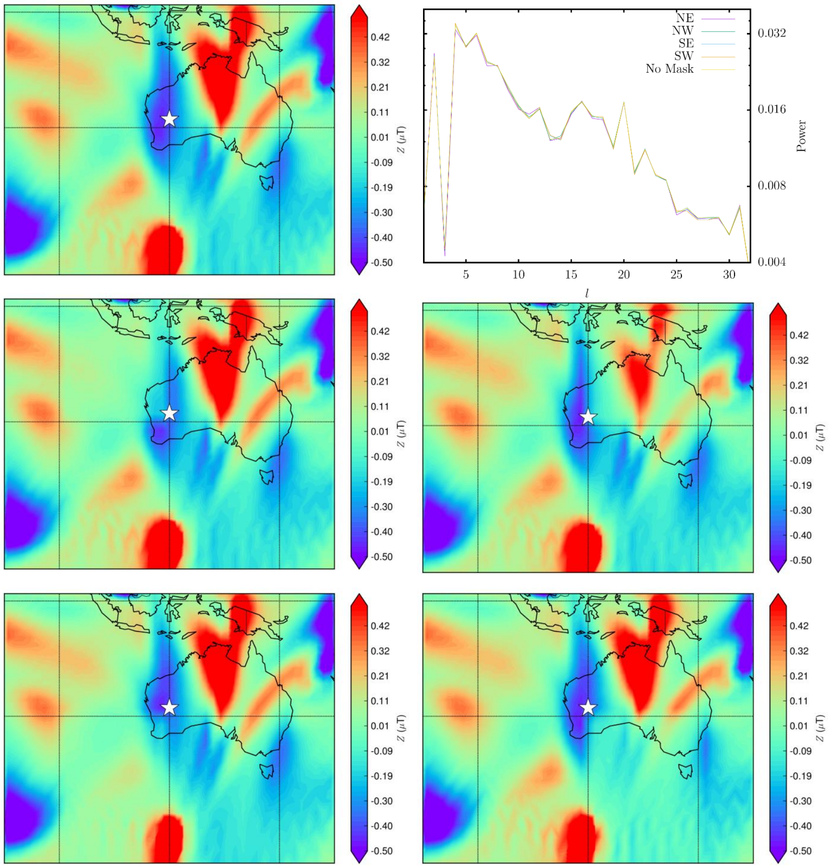

Supplementary Figure 7: Masking of magnetic features on the CMB in the simulation with $E = 5 \times 10^{-4}$, $Pm = 10$, $Ra = 350$ and $Rm = 450$ shown in Figure 6c of the main text and in Supplementary Figure 3. The top left panel shows the original field (left column of Supplementary Figure 3) with the extreme event location identified by a star. The Mercator maps below show the field with a mask applied to the north-west (NW) quadrant (top left , $30S - 0°\theta, 90 - 120°\phi$), north-east (NE) quadrant (top right, $30S - 0°\theta, 120 - 150°\phi$), south-west (SW) quadrant (bottom left, $S30 - 60S°\theta, 90 - 120°\phi$) and south-east (SE) quadrant (bottom left, $S30 - 60S°\theta, 120 - 150°\phi$). The quadrants tesselate the regions shown. The top-right panel shows the power spectrum $P(l) = (a/c)^{2l+4} \sum_{m=0}^{l}[(g_l^m)^2 + (h_l^m)^2]$ for the five different maps.

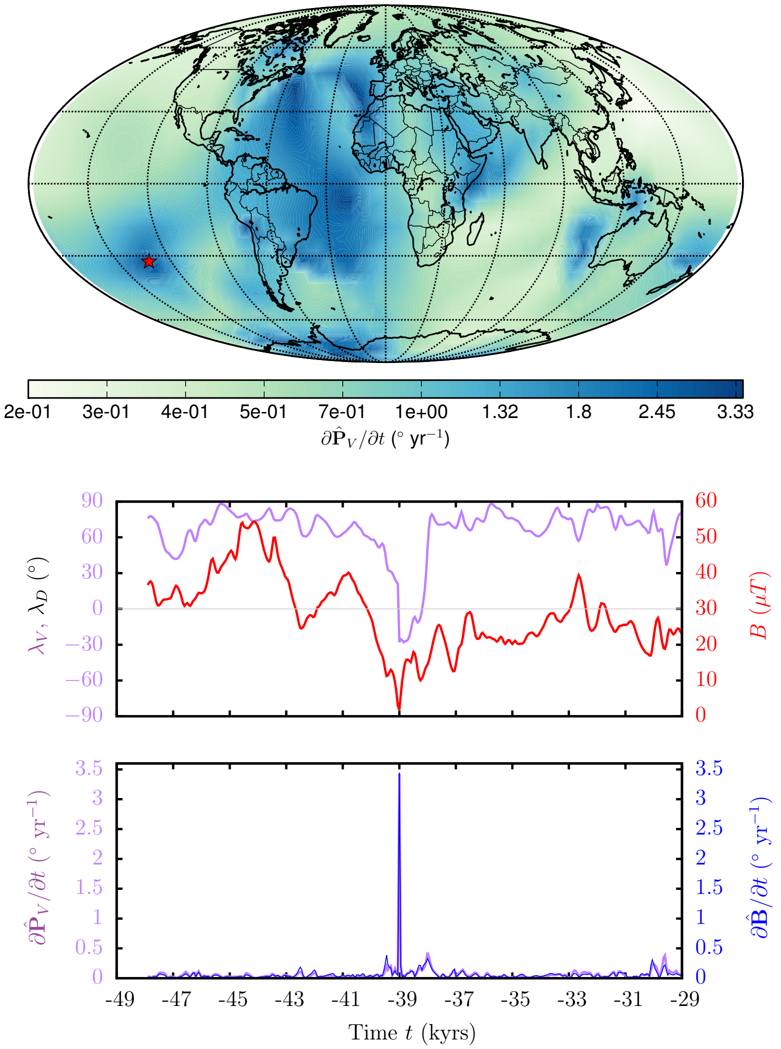

Supplementary Figure 8: Rapid directional changes in the observational field model LSMOD.2[1]. Top shows a Mollweide projection at Earth's surface of the largest change in VGP position, $\partial\hat{\mathbf{P}}_V/\partial t$, as a function of location in $^\circ yr^{-1}$. The red star shows the location of $(\partial\hat{\mathbf{P}}_V/\partial t)_{\mathrm{ex}}$. Bottom panel shows directional data at the location of $(\partial\hat{\mathbf{P}}_V/\partial t)_{\mathrm{ex}}$ over a 20 kyr period with the extreme event at the midpoint. Here the top row shows the latitude λ_V of $\hat{\mathbf{P}}_V$ (purple) and the field strength B (red); the bottom row shows $\partial\hat{\mathbf{P}}_V/\partial t$ (purple) and the rate of change of the field vector $\partial\hat{\mathbf{B}}/\partial t$ (blue).

E	Pm	Ra	$q^\star$	BC	Rm	τ	$\lambda_{\rm ex}(\hat{\mathbf{B}})$	$\phi_{\rm ex}(\hat{\mathbf{B}})$	$\partial\hat{\mathbf{B}}/\partial t$
0.00100	10	150	0.0	FTFF	261	329	-28	234	2.21
0.00100	10	70	0.0	FTFF	126	343	18	156	0.59
0.00100	10	90	0.0	FTFF	153	396	14	64	0.64
0.00001	10	225	2.3	FFFF	243	24	8	202	0.39
0.00001	10	225	2.3	FFFF	270	22	8	192	0.68
0.00012	10	150	0.9	FTFF	351	24	24	312	1.07
0.00012	10	300	0.0	FTFF	540	182	-22	160	0.92
0.00012	10	35	0.3	FTFF	108	195	-10	250	0.49
0.00012	10	35	0.9	FTFF	135	228	4	122	0.52
0.00012	10	450	0.0	FTFF	684	38	-28	280	7.28
0.00050	10	250	0.0	FFFF	386	232	-6	290	4.89
0.00050	10	350	0.0	FFFF	450	415	-28	120	9.12
0.00050	5	250	0.0	FFFF	225	431	0	4	3.55
0.00050	5	350	0.0	FFFF	252	607	-6	356	4.01
0.00050	5	400	0.0	FTFF	257	310	42	94	3.82
0.00050	5	450	0.0	FFFF	279	380	32	24	5.12

Supplementary Table 1: Runs used in this study. The Ekman number E, magnetic Prandtl number Pm, Rayleigh number Ra and amplitude of boundary heat flow heterogeneity $q^\star$ (=0 for homogeneous boundaries) are input parameters to the simulation along with the Prandtl number which is always set to unity. BC refers to the thermal boundary conditions used: FF is fixed flux; FT is fixed temperature; first two letters refers to the inner boundary; second two letters refers to the outer boundary. τ gives the length of the simulation in kyrs. The magnetic Reynolds number Rm is a simulation output. The remaining columns provide the latitude (in degrees), longitude (in degrees) and amplitude (in $^\circ$ yr^{-1}) of the maximum change in the field vector $\hat{\mathbf{B}}$. The simulations with $E = 5 \times 10^{-4}$ and 1.2×10^{-4} were originally published in ref. 2, the simulations with $E = 10^{-5}$ were published in ref. 3 and the simulations with $E = 10^{-3}$ were published in ref. 4.

Supplementary References

1. Brown, M., Korte, M., Holme, R., Wardinski, I. & Gunnarson, S. Earth's magnetic field is probably not reversing. *Proc. Natl. Acad. Sci.* **115**, 5111–5116 (2018).

2. Davies, C. & Constable, C. Insights from geodynamo simulations into long-term geomagnetic field behaviour. *Earth Planet. Sci. Lett.* **404**, 238–249 (2014).

3. Mound, J., Davies, C. & Silva, L. Inner core translation and the hemispheric balance of the geomagnetic field. *Earth Planet. Sci. Lett.* **424**, 148–157 (2015).

4. Sprain, C. J., Biggin, A. J., Davies, C. J., Bono, R. K. & Meduri, D. G. An assessment of long duration geodynamo simulations using new paleomagnetic modeling criteria (QPM). *Earth Planet. Sci. Lett.* **526**, 115758 (2019).
